# Celebrating oncology nursing: from adversity to opportunity. The Global Power of Oncology Nursing Conference held virtually on the 15th November 2022

**DOI:** 10.3332/ecancer.2023.1507

**Published:** 2023-02-09

**Authors:** Julia Downing, Gülcan Bağçivan, Kathryn Burns, Julia Challinor, Susanne Cruickshank, Martje de Villiers, Celia Diez de Los Rios de la Serna, Myrna Doumit, Nellie Kumaralingam, Mark Lodge, Lucia Robles Mendoza, Rima Saad Rassam, Vera Samba, Elaine Tomlins, Annie Young

**Affiliations:** 1 Makerere/Mulago Palliative Care Unit, Kampala, Uganda; 2 International Children’s Palliative Care Network, Durban 3624, South Africa; 3 International Society of Nurses in Cancer Care, Vancouver BC V6E 2V2, Canada; 4 Koç University School of Nursing, Istanbul, Topkapi 34010, Turkey; 5 Conference Coordinator, Budapest, Hungary; 6 University of California San Francisco, San Francisco, CA 94143, USA; 7 The Royal Marsden NHS Foundation Trust, London SW63 6JJ, UK; 8 Tshwane University of Technology, Pretoria 0183, South Africa; 9 Universitat de Barcelona, Barcelona 08007, Spain; 10 American University of Beirut, Beirut 1107 2020, Lebanon; 11 Maidstone and Tunbridge Wells NHS Trust, Maidstone ME16 9QQ, UK; 12 International Network for Cancer Treatment and Research, Oxford OX2 7HT, UK; 13 National Institute of Cancerology Mexico, Mexico City 14080, Mexico; 14 St Jude Children’s Research Hospital, Memphis, TN 38105, USA; 15 Cameroon Baptist Convention Health Services, Bamenda, Cameroon; 16 University of Warwick, Coventry CV4 7AL, UK

**Keywords:** nursing, cancer care, palliative care, health workforce, humanitarian settings, climate change

## Abstract

The Global Power of Oncology Nursing held their 3rd annual conference on ‘Celebrating Oncology Nursing: From Adversity to Opportunity’. The conference, held virtually, addressed three major nursing challenges: health workforce and migration, climate change and cancer nursing within humanitarian settings. Around the world, nurses are working in situations of adversity, whether due to the ongoing pandemic, humanitarian crises such as war or floods, shortage of nurses and other health workers, and high clinical demands leading to overwork, stress and burnout. The conference was held in two parts in order to take into account different time zones. Three hundred and fifty participants attended from 46 countries, with part of the conference being held in both English and Spanish. It was an opportunity for oncology nurses around the world to share their experiences and the realities for their patients seeking care and their families. The conference took the form of panel discussions, videos, and individual presentations from all six WHO regions and highlighted the importance of oncology nurses role in expanding beyond caring for individuals and their families, to tackle wider issues, such as nurse migration, climate change and care within humanitarian settings.

## Introduction

The Global Power of Oncology Nursing (GPON) 3rd annual conference (7-hour webinar) ‘Celebrating Oncology Nursing: From Adversity to Opportunity’ was held on the 15th November 2022 and addressed three major nursing challenges: health workforce and migration, climate change and cancer nursing within humanitarian settings. Around the world, nurses are working in situations of adversity, whether due to the ongoing pandemic, humanitarian crises such as war or floods, shortage of nurses and other health workers, and high clinical demands leading to overwork, stress and burnout. GPON brought together oncology nurses and other key stakeholders, particularly from low- and middle-income countries (LMIC), to share their experiences and the realities for their patients seeking care and their families. Panel discussions, videos, and individual presentations from all six WHO regions provided the global view of oncology nursing in the three adverse situations and the efforts these oncology nurses and their colleagues are making for amelioration.

## Background to the conference

The State of the World’s Nursing Report (SOWN) [1] published in 2020 emphasises the importance of investing in nursing through developing leadership, tackling retention and supporting nurses working in a range of settings. It highlights the importance of these actions in meeting the Sustainable Development Goal (SDGs) targets on health (SDG3), education (SDG4), gender equality (SDG 5) and decent work and economic growth (SDG8) [2]. The report was published during the global pandemic and at a time when the commitment to the provision of Universal Health Coverage (UHC) was highlighted [1, 3]. Whilst celebrating the work of nurses, the report underscores the unbalanced distribution of nurses around the world, the challenges that they face and the need to invest in education, create >6 million new nursing jobs by 2030 (many of which must be in LMICs), and strengthen nursing leadership [1]. SOWN also highlights key nursing roles in 21st-century health systems, particularly in achieving UHC, managing emergencies, epidemics and disasters and achieving population health and well-being (Table 1) [1]. All these issues are relevant for oncology nurses and impact on care delivery and the health of their patient populations everywhere. GPON’s vision, therefore, is to achieve UHC, including financial risk protection, access to quality essential health-care services and access to safe, effective, quality and affordable essential medicines and vaccines for all, in the context of cancer care (SDG 3.8).

## Nurse migration

With nearly 28 million nurses globally, nursing is the largest occupational group in the health sector, accounting for 59% of health professionals globally [1]. Migration and developing the nursing workforce are key issues globally. The global shortage of nurses is most acute (80%) in LMICs. Nurses migrate to higher income countries for many reasons including to increase their professional opportunities, optimise their earnings and support their families back home. Nurses’ migration has been a challenge for many years [4, 5], yet has been accentuated with the current global economic crisis and pandemic. Shortages in high-income countries lead to recruiting from LMICs, thus depleting the nursing workforce in countries where there are already glaring shortages. This wave did not spare oncology nurses who are being recruited to replace depleted staff in countries with comprehensive cancer treatment centres due to pandemic deaths, lure of high salaries for travelling nurses and nursing burnout [6].

Yet nurses deserve the opportunity to develop their practice in their local settings and function in a work environment where their specialised skills are recognised and remunerated appropriately. How do we address push and pull factors of migration, inequalities in nursing specialisation recognition and what does all this mean for us as nurses caring for patients with cancer? The International Centre on Nurse Migration [7] estimates that over the next 10 years we will need 10.6 million new nurses to address both the current shortage and those expected to retire during that time. Whilst an issue across the board for nurses, and all impacted by the recent pandemic [8] this is a particular issue within cancer care as there is often a lack of basic and specialised oncology nurse training and career development within the field globally [9].

**Figure 1.**
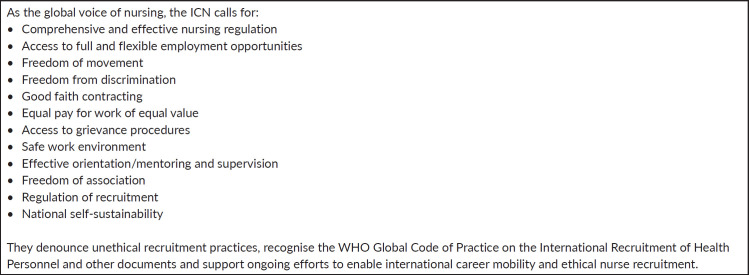
ICN position and recommendations on international career mobility and ethical nurse recruitment [10].

In its position statement on international career mobility and ethical nurse recruitment, the International Council of Nurses (ICN) [10] reiterates the importance of having a healthcare workforce with an adequate number of qualified, motivated and well-trained nurses. The ICN recognises that career mobility is essential for nurses to further their professional development and build nursing services within their home country. However, the ICN also notes the challenges of global nurse migration and calls for a range of recommendations in order to address the challenges (Figure 1), many of which are echoed by nursing associations around the world. There is a lack of literature on the worldwide recruitment of oncology nurses specifically [11], yet there is global recognition of the challenge [12] and of the need to strengthen the global oncology workforce in LMICs [13]. Across the cancer care continuum, the impact of nurse migration has been reported, e.g., in Jamaica with regard to palliative care [14], in the UK regarding breast care [15] and globally [11].

## Climate change

Alongside issues of migration, climate change is posing an increasing and ongoing challenge to all of us around the world. Climate change impacts every area of cancer care [16, 17], and indeed all aspects of health care. In 2021, The Lancet published a joint comment on the climate emergency calling for urgent action from world leaders [18]. Individuals with cancer are susceptible to the disruption of the complex and integrated health-care systems caused by climate change events [17]. The Lancet article authors reiterate that our health and that of future generations depend on all of us acting now. We know that climate change is a public health crisis, and one that we as nurses cannot fail to act upon. Dickman *et al* [19] state that climate change increases exposure to known carcinogens leading to increased incidence of patients with cancer, thus, oncology nurses must be involved in finding solutions to climate change. Oncology nurses *play an essential role in educating patients on environmental health, identifying sustainable healthcare practices and acting as environmental stewards and advocates* (p 109). The authors build upon the paper by Butterfield *et al* [20] that highlights nursing’s pivotal role in global climate action. The authors recognise that nurses possess an unparalleled collective potential to change the trajectory of climate change given their early involvement in the climate debate and the trust that they gained among people most vulnerable to climate change. The authors make five recommendations for nurses: 1) leapfrog nurses into leadership roles, 2) give nurses the right skills, 3) promote activism and advocacy, 4) think global and 5) address climate change as a moral problem. Much has been written in the past 5–10 years with regard to climate change and health and there is robust science on climate and health and many calls for us as cancer nurses to act, and to act now [16, 21–26]. The ICN calls for each of us to advocate for policies that promote the reduction of healthcare waste and ensure sustainable waste management, to engage in committees and policy making, and to empower individuals to make healthy lifestyle changes [26].

## Oncology nursing in humanitarian settings

Alongside this, issues of providing cancer care in humanitarian settings are at the forefront of global oncology discussions. It is estimated that in 2022, 274 million people will need humanitarian assistance and protection [27]. Concerns highlighting the global trends and humanitarian crisis in multiple countries thought to dominate 2022, include COVID-19, climate change, conflict, hunger, displacement and gender inequalities. The International Rescue Committee has identified political tension and conflict in a range of countries including Ukraine, Sudan, Syria, Somalia where there is also drought, Myanmar, Democratic Republic of Congo, South Sudan, Nigeria, Yemen, Ethiopia and Afghanistan [28]. The Global Humanitarian Assistance Report 2022 [29] highlights the unprecedented challenges of the humanitarian support system with more countries enduring crises for longer. The authors address the interconnections between responses to climate change and humanitarian crises and review efforts to improve the effectiveness and efficiency of the humanitarian response. Cancer prevention and treatment in humanitarian settings is recognised as an urgent and unmet need [30], thus highlighting and identifying the need for cancer care during war and conflict [31], the need for cancer care for refugees and displaced populations [32], the complexity of care delivery in these settings with limited availability of resources and a lack of accurate data on which to base policy decisions [33]. There is also a need for cancer prevention and treatment in humanitarian settings [32] and the provision of palliative care in humanitarian settings [34, 35].

## The GPON conference 2022

The GPON conference [36] was held on Tuesday 15th November 2022 as part of London Global Cancer Week (LGCW) [37], which aims to raise awareness about global cancer to create a catalyst for change. Since 2020, after a successful inaugural virtual conference during the pandemic, GPON is continuing with virtual conferences being held annually to facilitate accessibility for global attendance and is organised by a group of international adult and paediatric oncology nurses. GPON 2022 was a free-to-attend virtual event which primarily focused on cancer care and nursing in LMICs and combines presentations and participation from caregivers, patients, survivors and industry partners from around the world [36]. This year, the conference was held in two parts – the day session, aimed towards Europe and the East from 10.00–15.00 GMT, and an evening session aimed at the Americas from 20.00–22.00 GMT. A total of 350 participants attended the conference from 46 countries, with 255 attending the day session and ~95 attending the evening session. The day session was held in English whilst the evening session was bilingual – English and Spanish. The conference was organised into a range of plenary presentations, panel discussions and video presentations, with 30 facilitators taking part from around the world including: Afghanistan, Argentina, Bhutan, Canada, Cameroon, Colombia, Ghana, Lebanon, Mexico, Nigeria, Pakistan, Panama, Peru, The Philippines, Somaliland, Spain, Syria, Turkiye, the Turks and Caicos, Uganda, Ukraine, the United Kingdom and the United States of America. With a theme of ‘From Adversity to Opportunity’, the conference focused on three main themes: 1) the nursing workforce and migration, 2) oncology nursing in humanitarian settings and 3) oncology nursing and climate change, with the first and third themes being explored in both parts of the conference.

The programme also included reflections on oncology nursing from around the world. The Dorcas Foundation shared about the worries and the hopes of paediatric oncology nurses in Nigeria. Lucia Robles Mendoza along with other colleagues from the National Cancer Institute in Mexico City spoke about what they liked best about being professional oncology nurses, challenges and their motivation to do this work. Aprille C Banayat from Cancer Warriors Foundation shared about oncology nursing in the Philippines and a video interview by well-renowned oncology nurse Luz Esperanza Ayala from Bogotá, Colombia, has been shared on the GPON website.

### The nursing workforce and migration

The keynote address on health workforce and oncology nursing in the Eastern Mediterranean region was given by Dr Fethiye Gulin Gedik, the Co-ordinator, Health Workforce in the WHO Regional Office for Eastern Mediterranean (EMRO). She has extensive experience globally and in the WHO regional and head offices and has worked with health workforce issues around the world. Dr Gedik started the conference by setting out the importance of workforce planning, the need for developing the nursing workforce, and in particular in relation to cancer care within the EMRO region. She highlighted the important work that nurses do and also the challenges that they face, and the inequities experienced in various parts of the region. She clearly set the scene for discussions around migration and strengthening and empowering the oncology nursing workforce.

Next, keynote speaker Professor James Buchan from Australia, working with the World Bank/WHO talked about where countries such as the UK are reliant on recruiting international nurses in order to fill their nursing posts. There has been much discussion around this issue, and that by bringing these nurses to the UK, the lack of nurses in their home countries is heightened. However, as outlined above, there are methods of ethical recruitment, and building and strengthening nursing in LMICs by providing opportunities for professional development and learning, which will enable them to strengthen nursing back home. Professor Buchan was able to share his extensive experience and lessons learnt, which were both enlightening and thought-provoking regarding nurse migration for both countries where nurses migrate from and where nurses migrate to.

Then a panel discussion on migration was held with oncology nurses from Turkiye (Professor Sevilay Senol Celik), Ghana (Enyo Bosumprah), Lebanon (Rima Saad) and the Philippines (Melvin Miranda), facilitated by Dr Gülcan Bağcivan from Turkiye. Whilst working in multiple countries, regions and differing political climates, some of the issues raised were similar – all speakers shared the increase of oncology nurses’ migration in their countries. Besides the speciality challenges (low enrolment, low retention, lack of training and burnout), in many LMICs, oncology nurses struggle with poor work conditions, lack of job stability, lack of human and material resources to deliver safe care. In addition, political instability and failing economies impose drastic living conditions for oncology nurses and their families. These reasons push oncology nurses to look abroad for better job conditions, more opportunities for professional development and safer work and living environments. The panellists described the consequences of migration including worsening of nurses’ shortage in the home country and the psychological effects on the departing and remaining nurses as well as on the oncology patients who bonded with their nurses. However, the panellist noted that migration enabled nurses to sharpen their skills, support their families and give back to their country. The panellists also highlighted strategies to reduce the impact of migration on the nursing care being provided in-country such as developing training and practice initiatives for nurses in their home countries, and facilitating collaborations between local institutions and world-class oncology centres.

Dr Myrna McLaughlin-Anderson, from the Ministry of Health Panama and the University of Panama continued this discussion in the evening GPON session. She mentioned the importance of utilising education to strengthen oncology nursing. She discussed the Pan American Health Organization’s (PAHO) response to the increasing burden of NCDs including cancer. Alongside promoting public awareness, reducing risk, raising taxes on tobacco, immunisation and screening, PAHO is committed to strengthening the oncology nursing workforce. Dr McLaughlin-Anderson emphasised the capabilities of all nurses of leading and engaging in meaningful roles to address workforce and health equity issues, depending on their individual interest, skills and opportunities. Lucia Robles Mendoza from the National Cancer Institute in Mexico City shared with the audience the challenges of oncology nurses’ migration in the region, and the existing strategies to mitigate the impact of these challenges, and to support oncology nurses in their career development. We also heard from Heidi Bludau from Vanderbilt University about research undertaken in the Czech Republic from looking at the experience of migrant nurses who had worked across Europe and the Middle East. Many motivators of nurses’ migration were related to increasing their personal and professional worth, self-development and better salaries and work conditions. Nevertheless, in their new settings, particularly in other countries in Europe, the migrating nurses struggle with language, discrimination and the high-cost of living.

### Oncology nursing in humanitarian settings

In recent years, the importance of oncology nursing within humanitarian settings gained attention. Our colleagues from Ukraine shared a video highlighting strategies for addressing paediatric oncology nursing during the war in Ukraine. We heard from Oksana and Iryna, nurses at the paediatric oncology clinic, West Ukrainian Specialised Paediatric Medical Centre in Lviv, thanks to the co-ordination of Dr Roman Kizyma. They described the management of patients during air raids at the hospital and the transfer of paediatric oncology patients to the bomb shelter to ensure safety and continuity of treatment. However, the environment is challenging with a lack of emergency services, electricity, light and water. Nevertheless, the oncology nurses do their best and continued the care of the children by extending their roles and seeking resources to ensure that the children and families are being cared for. With the help of charities such as Tabletochki and Safer Ukraine, many children with cancer have been evacuated to nearby countries to continue their treatment, e.g. Poland and across Europe and the UK as well as the US, and Canada. The video was a powerful and moving account reflecting the reality of heroic measures these paediatric oncology nurses are taking to provide care amidst incredible adversities, a true demonstration of resilience and devotion to care for children with cancer and their families.

The video was followed by a panel discussion with oncology nurses from Afghanistan (Anonymous), Syria (Amaal Safadi), Uganda (Mariam Ndagire) and Cameroon (Vera Samba) facilitated by Dr Myrna Doumit from Lebanon. The discussion focused on strategies for addressing oncology nursing in crisis situations, with participants working in a range of humanitarian settings. Each was facing distinct challenges since the humanitarian situations were long standing and ranged from providing cancer care to refugees to working in the countries with existing humanitarian crises. Whilst the oncology nurses are managing staff shortages, patient/family transportation issues getting to appointments and political turmoil causing stress on oncology professionals, patients and families and supply chains for essential medicines and cancer services, it was clear throughout that as oncology nurses they have learnt to be resilient and to think out of the box, to look at ways to overcome challenges, and to support each other through the stress and burnout that they are experiencing.

Developing oncology nursing in a country that has been experiencing a humanitarian crisis is a challenge, and we heard from Yangden Paki from the home-based palliative care programme at Jigme Dorji Wangchuck National Referral Hospital in Bhutan about this. She described the lack of training within the country, of clinical placement sites, and access to the overall cancer control spectrum including prevention, screening, early diagnosis, treatment and palliative care. Learning from others in the region is important along with sharing resources and success stories and how these may be applied in Bhutan. She was able to demonstrate the development of oncology nursing over the past 5–10 years and how they are overcoming challenges and moving forward together particularly in much needed palliative care.

### Oncology nursing and climate change

Climate change is an issue that impacts all of us and requires collective actions. Videos and presentations from throughout the world illustrated the impact of climate change on oncology patient care (Edna Adan Ismail – Somaliliand, Dilnasheen Safdar – Pakistan, Latoya Silveri – Turks and Caicos). The recent extreme flooding in Pakistan, impeded the care of children with cancer. The continuing drought in Somaliland and the recent hurricane in the Turks and Caicos deeply impacted health and nursing care. These are countries least able to cope with disaster. In Somaliland, a country where there is virtually no cancer care, the ongoing drought is causing further starvation in rural areas, increased neonatal mortality and an end to a nomadic way of life for many people. In the day session of the conference, Ann Keen from the UK, a nurse and former member of parliament, emphasised the power of the nurse. She told us that we should never underestimate the voice and power of the nurse, especially when we speak together with a strong united voice. As nurses we have a major role to play in tackling climate change issues, and we need to work together to do this. She emphasised the challenge to cancer care of climate change and ways that we as nurses can help to mitigate these challenges. She mentioned that it is not about if we want to get involved, but that we must; we have an imperative as cancer nurses to get involved in the climate change debate, to be catalysts for change and have a responsibility to bring about change. Oncology nurse, Wanda Martin from the University of Saskatchewan Canada then continued the discussion with regard to the impact of climate change on oncology nursing. She explored some of the issues raised, sharing examples from her experience in addressing public health challenges for applied outcomes with a focus on health equity, food systems and climate change. All of these issues are interlinked and climate change also impacts health equity and food systems. Based on her research and experience in this area, Wanda Martin provided practical advice as to how, we, as oncology nurses can positively address climate change.

Following these calls to action in the day session of the conference, Dr Leticia Noguiera from the American Cancer Society spoke to the audience in the Americas about the fact that climate change was not just happening ‘over there’ in the Europe, Africa and the East. She detailed the source of carcinogens due to climate change including the world’s reliance on fossil fuels and carcinogenic fossil fuel waste. She specifically pointed out interruption in access to care due to extreme weather events such as hurricanes, emphasising that ‘disasters are the result of social constructions, economic models, and political choices’.

We also heard from Dr Marisa Gaioli, a paediatrician from Argentina who is Chair of her hospital committee on climate change at the Hospital of Pediatrics S.A.M.I.C. Professor Dr Juan P. Garrahan and oncology nurse Giovanna Rosada from the Clinica Auna Oncosalud, Peru in the evening. Both speakers further explored the role of climate change and oncology care, the effect of climate change on cancer and the work of oncology nursing. Dr Gaioli exhorted oncology nurses to *Truthfully inform patients and their families about the effects of climate change on health to avoid eco-anxiety*. Ms Rosada mentioned *Our objective is to reduce contamination to a minimum, raising awareness among those responsible for choosing the least harmful materials and products and adopting technical measures aimed at reducing contamination of the work environment and food products.*

The evidence is clear – climate change impacts on cancer control, which impacts on the work of oncology nurses. We therefore have no choice and must get involved in the climate change debate, making changes and trying to lessen the ongoing effects of climate change in general, but for our patients and families as well as survivors!

## Cycling to China

The inspirational presentation of the day session was from Luke Grenfell-Shaw, a CanLiver (an individual facing the challenges and uncertainties of cancer on a daily basis, yet acknowledging that we all can live with cancer – richly and fully) who set out to cycle from Bristol in the UK to China to raise awareness of cancer in younger people [38]. He was diagnosed in 2018 with a rare and aggressive metastatic sarcoma at the age of 24. He underwent chemotherapy, surgery and radiotherapy yet did everything he could to keep living richly and fully, undertaking the Bristol marathon halfway through chemotherapy. He set out to ‘live his dream’ cycling from Bristol to Beijing on a tandem bike, joined by other CanLiver’s, rewriting the narrative of what is possible with a cancer diagnosis. He shared his story of how he cycled 27,000 km with over 300 people joining him on the back of his tandem across Europe and Asia, but was unable to finally cross into China due to COVID – just 3,300 km short of Beijing. He returned to the UK and pedalled the remaining distance finally completing the ride on the 19th June 2022. Luke shared his powerful and inspirational story and as one participant commented: *a very powerful and breath-taking story indeed! This is what keeps us, oncology nurses, striving to offer our best care possible, Hats off Luke!.*

## GPON awards and launch of Continulus Pocketbook

There were four categories of awards given at the conference by GPON. Applications for the awards had been open for 2 months, and individuals were able to self-nominate or be nominated for the awards. The awards were sponsored by UKONS, the UK Oncology Nursing Society (ONS) and the recipients of the awards were announced at the conference (Table 2).

The GPON 2022 conference was also the opportunity to launch the new ***Pocketbook of Cancer Care Nursing*** [39]*.* The pocketbook is being produced by Continulus in partnership with GPON and the International Society of Nurses in Cancer Care (ISNCC) [39]. There are over 120 lectures on the core topics of cancer care nursing delivered by experts from around the world. These are available for access online at any time, from any place and are free of charge. New content is being added all the time.

## Take away messages

At the close of each part of the conference, three key messages from the sessions were shared.

The key messages from the day session were:

1. Nurses still care daily for people with cancer in crisis zones despite the huge risks.
2. All countries have ethical codes for health care migration – individual nurses need to speak up if not followed.
3. Nurses must speak up about climate change – we have to take collaborative action to mitigate its impact.

The key messages from the evening session were:

1. Nurses in government positions make a big difference.
2. Nurse migration is complex and it is not all about money.
3. Climate change is everyone’s business.

There was a strong feeling that ‘we are on this walk together’, that we need to collaborate, and walk hand-in-hand as we travel this journey together to provide oncology care in humanitarian settings, tackle climate change and address the challenges of oncology nurse migration.

## Conclusion

The conference highlighted the fact that as oncology nurses, care expands beyond individual interventions for our patients and their families, to tackle wider issues, such as nurse migration, climate change and how we provide care in humanitarian settings. If we stand together as oncology nurses, speaking with one voice and working collaboratively we can and will make a difference, but we need to be prepared and educated to take that stand, to use our voice, and to speak out and make that difference.

## Conflicts of interest

The authors declare that they have no conflicts of interest.

## Figures and Tables

**Table 1.** Nursing contribution to the triple billion targets [1].

Nurses as part of the multidisciplinary team	UHC	Front-line providers of primary care Preventing and treating wide range of communicable and noncommunicable diseases (NCDs) Offering care across the life course, from birth to death
	Emergencies, epidemics and disasters	Delivering care for clinical emergencies Responding to epidemics, disasters and humanitarian crises Recognising life-threatening conditions and performing life-saving procedures
	Health and well-being	Addressing the social determinants of health through collaborative action Addressing and treating the impacts of climate change Ensuring access for vulnerable groups, including women and youths

**Table 2.** Recipients of the GPONs Awards 2022.

Two GPON Rising Star Awards Early LMIC oncology career nurses who demonstrate exceptional nursing-related practice, education or leadership.	Recipients Amish Kale – Tata Memorial Center, Mumbai, India Ruth Huamani – National Institute of Neoplastic Diseases (INEN), Lima, Peru
**Two GPON Outstanding Contribution to Cancer Nursing Awards** Projects or work that has made a difference in an LMIC or region/area where there is concern	**Recipients** Anku M Frances – Komfo Anokye Teaching Hospital, Kumasi, Ghana Gloria Ceballo – Hospital del Niño José Renán Equivel, Panama City, Panama
**Two GPON Master of Nursing Awards** Individuals who have made a significant contribution to cancer nursing in a LMIC	**Recipients** Vera Samba – Mboppi Baptist Hospital, Douala, Cameroon Dilnasheen Safdar – National Institute of Blood Diseases and BMT/University of Child Health and Children Hospital, Pakistan
**One GPON Team/Unit/Ward Award ** A multidisciplinary team of four or more individuals, must include a nurse, who have made a significant contribution to cancer care	**Recipients** Nursing Management Team – INEN, Lima, Peru

## References

[1] World Health Organization (2020). State of the World’s Nursing 2020: Investing in Education, Jobs and Leadership.

[2] United Nations Development Programme (2022). What are the sustainable development goals. https://www.undp.org/sustainable-development-goals.

[3] World Health Organization (2012). Universal health coverage. https://www.who.int/news-room/fact-sheets/detail/universal-health-coverage-(uhc).

[4] Yeates N (2010). The globalization of nurse migration: policy issues and responses. Int Labour Rev.

[5] World Health Organization (2010). WHO Global Code of Practice on the International Recruitment of Health Personnel.

[6] Galassi A, Challinor J (2015). Strengthening the oncology nurse workforce in low-income and middle-income countries. Lancet Oncol.

[7] American Journal of Nursing (2021). NewsCAP nursing workforce crisis looms with 4 million nurses retiring by 2030. Am J Nurs.

[8] Buchan J, Catton H (2020). COVID-19 and the international supply of nurses: report for the International Council of Nurses. https://www.icn.ch/system/files/documents/2020-07/COVID19_internationalsupplyofnurses_Report_FINAL.pdf.

[9] Nwozichi CU, Ojewole F, Oluwatosin AO (2016). Understanding the challenges of providing holistic oncology nursing care in Nigeria. Asia Pac J Oncol Nurs.

[10] International Council of Nurses (2019). Position statement: international career mobility and ethical nurse recruitment. https://www.icn.ch/system/files/documents/2019-11/PS_C_International%20career%20mobility%20and%20ethical%20nurse%20recruitment_En.pdf.

[11] Challinor JM, Alqudimat MR, Teixeira TA (2020). Oncology nursing 2. Oncology nursing workforce: challenges, solutions and future strategies. Lancet Oncol.

[12] Al-Sukhun S, Lima Lopes G, Gospodarowicz M (2017). Global health initiatives of the international oncology community. Am Soc Clin Oncol Educ Book.

[13] Mathew A (2018). Global survey of clinical oncology workforce. J Glob Oncol.

[14] Edwards RL, Patrician PA, Bakitas M (2021). Palliative care integration: a critical review of nurse migration effect in Jamaica. BMC Palliat Care.

[15] Breast Cancer Care Now Breast Cancer Care’s Response to the Migration Advisory Committee’s Consultation on Their Review of the Shortage Occupation List.

[16] Kagan SH (2022). Guest Editorial. Treating our malignant climate: global health, healthy climate and cancer nursing. Cancer Nurs.

[17] Editorial (2021). Climate crisis and cancer: the need for urgent action. Lancet Oncol.

[18] Atwoli L, Baqui AH, Benfield T (2021). Call for emergency action to limit global temperature increases, restore biodiversity, and protect health. The Lancet.

[19] Dickman E, Backler C, Berg CD (2022). Climate change and oncology nursing: a call to action. Clin J Oncol Nurs.

[20] Butterfield P, Leffers J, Diaz Vasquez M (2021). The future of nursing: nursing’s pivotal role in global climate action. BMJ.

[21] Becze E (2022). Climate change is contributing to the cancer burden, and nurses must take action. ONS Voice. https://voice.ons.org/news-and-views/climate-change-is-contributing-to-the-cancer-burden-and-nurses-must-take-action.

[22] Alliance of Nurses for Health Environments (2022). https://envirn.org/jedi/.

[23] Nurses and Nurse Practitioners of British Columbia (2022). The Role of Nursing in Climate Change. Actions for Nurses in a Changing World.

[24] Sayre L, Carpenter H (2010). Climate change and human health. The role of nurses in confronting the issue. Nurs Admin Q.

[25] Ford M (2021). Why nursing needs to act now on sustainability. Nurs Times.

[26] International Council of Nurses (2018). Position statement: International Council of Nurses calls for increased nursing leadership to combat effects of climate change on health. https://www.icn.ch/news/international-council-nurses-calls-increased-nursing-leadership-combat-effects-climate-change#:~:text=As%20the%20global%20voice%20of,pollution%2C%20degradation%20and%20destruction.%E2%80%9D.

[27] Bloxham L (2021). Concern worldwide. Six humanitarian crises to watch out for in 2022. https://www.concern.org.uk/news/six-humanitarian-crises-watch-out-2022.

[28] International Rescue Committee (2021). https://www.rescue.org/article/top-10-crises-world-cant-ignore-2022.

[29] Urquhart A, Girling F, Mason E (2022). Global humanitarian assistance report 2022. Development initiatives, global humanitarian assistance. https://devinit.org/resources/global-humanitarian-assistance-report-2022/#downloads.

[30] Alawa J, Maiky C (2019). Cancer prevention and treatment in humanitarian settings: an urgent and unmet need. Lancet Oncol.

[31] El Sayed R, Mukherji D, Al-Shamsi HO, Abu-Gheida IH, Iqbal F (2022). Cancer care during war and conflict. Cancer in the Arab World.

[32] El Saghir NS, Perez de Celis ES, Fares JE (2018). Cancer care for refugees and displaced populations: middle east conflicts and global natural disasters. Cancer care for refugee and displaced populations. Am Soc Clin Oncol Educ Book.

[33] Saab R, Slama S, Mansour A (2022). Cancer control eastern mediterranean region special report. Chapter 6: Cancer Care in Humanitarian Crises.

[34] Nouvet E, Sivaram M, Bezanson K (2018). Palliative care in humanitarian crises: a review of the literature. J Int Humanit Action.

[35] WHO (2018). Integrating Palliative Care and Symptom Relief into Responses to Humanitarian Emergencies and Crises: A WHO Guide.

[36] https://theglobalpowerofoncologynursing.com/gpon-2022/.

[37] https://www.lgcw.org.uk.

[38] https://www.bristol2beijing.org/thejourney.

[39] https://www.continulus.com/library/?collection=cancer+care+nursing&chapter=palliative+care.

